# Response of Acala Cotton to Nitrogen Rates in the San Joaquin Valley of California

**DOI:** 10.1100/tsw.2001.334

**Published:** 2001-11-20

**Authors:** R.B. Hutmacher, R.L. Travis, R.L. Nichols, D.E. Rains, B.A. Roberts, B.L. Weir, R. M. Vargas, B. H. Marsh, S. D. Wright, D. S. Munk, D. J. Munier, M. P. Keeley, F. Fritschi, R. L. Delgado, S. Perkins

**Affiliations:** Shafter Research and Extension Center, University of California, Shafter 93263, USA

**Keywords:** nitrogen, nitrogen management, residual nutrients, fertility, fertilizer response, cotton, Gossypium hirsutum L., soluble nutrients, nitrate, nitrate leaching, yield response, groundwater, groundwater nitrate, fertilizer optimization

## Abstract

The responses of Acala cotton (Gossypium hirsutum L.) in California to a range of applied nitrogen (N) treatments were investigated in a 5-year, multisite experiment. The experiment’s goals were to identify crop growth and yield responses to applied N and provide information to better assess the utility of soil residual N estimates in improving fertilizer management. Baseline fertilizer application rates for the lowest applied N treatments were based on residual soil nitrate-N (NO_3_-N) levels determined on soil samples from the upper 0.6 m of the soil collected prior to spring N fertilization and within 1 week postplanting each year. Results have shown positive cotton lint yield responses to increases in applied N across the 56 to 224 kg N/ha range in only 41% (16 out of 39) of test sites. Soil NO_3_-N monitoring to a depth of 2.4 m in the spring (after planting) and fall (postharvest) indicate most changes in soil NO_3_ occur within the upper 1.2 m of soil. However, some sites (those most prone to leaching losses of soluble nutrients) also exhibited net increases in soil NO_3_-N in the 1.2- to 2.4-m depth zone when comparing planting time vs. postharvest data. The lack of yield responses and soil NO_3_-N accumulations at some sites indicate that more efforts should be put into identifying the amount of plant N requirements that can be met from residual soil N, rather than solely from fertilizer N applications.

## INTRODUCTION

There are several incentives for considering adjustment of the N management practices of cotton and other California crops. With cotton, mid- and late-season N management has an impact on the crop's progress toward cutout, readiness for defoliation and harvesting ease. High N levels during bloom and early boll filling can also promote vegetative development at the expense of fruit retention under some conditions[1,2]. High N levels in cotton can delay harvest, can have a negative impact on defoliation costs and efficacy in leaf removal, and can increase problems with some late-season pests, e.g., silverleaf whiteflies and aphids, which can influence lint quality[3]. Recent increases in energy costs, which constitute a large part of N fertilizer production costs, have been passed on as increases in N fertilizer cost. An additional area of concern is the fate of N applied in excess of plant requirements. If crops grown in the rotation sequence do not have deep enough roots to intercept applied and residual N, its eventual movement through the soil profile can result in $NO_3$-contamination of groundwater[4]. The potential for $NO_3$- contamination is a health concern for drinking-water supplies in many parts of California as well as in other regions and can also impact other municipal and agricultural groundwater uses.

## MATERIALS AND METHODS

The experiments were conducted on the University of California (UC) West Side and Shafter Research and Extension Centers and at five to six grower fields per year in Fresno, Madera, Merced, Kings, Tulare, and Kern Counties in California's San Joaquin Valley. Some field sites were utilized for multiple years (about one third of the field sites over the 5-year period), while the remaining sites were newly chosen each season due to grower decisions on crop rotations. The UC research center sites had plots 4 rows in width by approximately 90-m long each year, while grower field sites were 6 or 8 row plots ranging from 200 m to over 450 m in length. Four field replications in a randomized complete block were used at all experiment locations.

Four basic N fertilization treatments were established at each site annually. The application rate was equal to the desired N treatment level in kg N/ha (either 56, 112, 168, or 224 kg N/ha), minus the calculated soil residual N value in kg $NO_3$-N/ha determined in the upper 0.6 m of the soil profile. Residual N levels were calculated using soil samples collected from the upper 0.6 m of soil within 4 to 7 days after planting, prior to any N fertilizer applications. If the initial amount of soil residual $NO_3$-N was greater than 56 kg $NO_3$-N/ha, the residual value was used as the baseline for the 56 kg N treatment. All other treatments were added in 56-kg increments after deducting the N present in the baseline treatment. Soil $PO_4$-P and exchangeable-K were also tested on soil samples, and fertilizer applications were made as necessary to ensure that soil P and K levels were nonlimiting to yield in this N experiment.

In 1996, four treatments of 56, 112, 168, and 224 kg N/ha were applied in late May (prior to the first postplanting irrigation), and in three supplemental treatments (in which 56, 112, or 168 kg N/ha was initially applied). A second N application of 56 kg N/ha was applied in June just prior to the second irrigation. In 1997 through 2000, the experiments were simplified to four basic treatments (56, 112 168, and 224 kg N/ha) due to the lack of crop growth and yield responses to split-application treatments used in 1996 and grower requests for simplified field applications.

Soil samples in all plot locations were collected for several purposes. Initially each year, samples were collected from six locations per field replication in the low N application treatment at each site. These samples were collected in 0.3-m increments to a depth of 0.6 m, composited within each field replication, and air dried at 35 to 40°C, and analyzed for $NO_3$-N[6]. In addition, for the purposes of evaluating $NO_3$- movement, soil samples were also collected to a depth of 2.4 m, twice annually, in all plots using a power-driven, soil-core sampling device with a 4.45-cm diameter tube. The two incidences for this deeper soil sampling were: (1) within 14 to 24 days after planting and (2) within 20 days after annual harvest. Each of three replicate plots within each treatment at each location was sampled in 0.3-cm increments to a depth of 1.2 m and in 0.6-m increments to an ending depth of 2.4 m, resulting in six separate samples per sample hole. Four locations within each plot were sampled in the first, second, and third years of the study, and three locations per plot were sampled in years four and five. The soil samples were kept refrigerated (2 to 4°C) until subsamples were collected for specific analyses. Separate subsamples were collected to evaluate gravimetric soil-water content and to provide subsamples to collect 2 N KCl extracts as well as air-dried soil samples. A 2 N KCl extract on the soil samples was used to determine soil $NO_3$-N and $NH_4$-N[5]. A separate subsample air dried at 35 to 45°C was prepared from a composite of the three to four sample locations for each depth within each plot and subsequently analyzed for $NO_3$-N, plus $PO_4$-P, $NH_4$+ acetate exchangeable-K and other nutrient, pH, or salinity analyses as each site required[6]. Bulk density was determined on 4.45-cm diameter soil-core samples collected at 0.15-m increments from three field replications per research site using only the postharvest soil samples.

Plant petiole samples were collected at intervals during each growing season at all sites to monitor for adequacy of N, P, and K. Petiole data will not be discussed for this paper, but will be discussed in later papers. Three replicate samples of irrigation water samples were collected at least monthly at each experiment site and analyzed for $NO_3$-N[5]. At all locations, seed cotton was mechanically harvested using commercial-type spindle pickers. Seed cotton yields were weighed in the field and 2.5-kg subsamples taken for determination of moisture content. The seed cotton was ginned at the UC Shafter Research and Extension Center Research Gin, and the lint, seed, and trash contents were determined. Lint and seed yields were calculated and adjusted for moisture content. Although fiber quality data will not be discussed in this paper, subsamples from each field sample were sent for high-volume instrumentation (HVI) analyses.

## RESULTS AND DISCUSSION

This report will focus mostly on (1) basic descriptions of soil $NO_3$-N status at the field plot sites in the immediate postplanting period each year, (2) crop yield responses to applied plus residual N, and (3) a limited discussion of deep soil sampling N data and average calculated values for changes in soil $NO_3$-N during growing seasons.

### Initial Soil $NO_3$-N Levels

The soil $NO_3$-N levels found in the upper 0.6 m of the soil profile within a few days postplanting covered a wide range of levels each year of the study. N as $NO_3$-N in the upper 0.6 m of the soil profile ranged from 77 to 251 kg N as $NO_3$-N/ha in 1996, 48 to 158 kg N as $NO_3$-N /ha in 1997, 40 to 114 kg N as $NO_3$-N/ha in 1998, 40 to 271 kg N as $NO_3$-N/ha in 1999, and 39 to 161 kg N as $NO_3$-N/ha in 2000 (see Table 1). Although not true in all cases, sites with relatively low residual soil $NO_3$-N in the upper 0.6 m of the soil profile generally were in cotton following either cotton or small grains, while moderate to high residual soil $NO_3$-N levels were more typically in cotton grown following field corn (for silage or grain), processing tomatoes, or forage alfalfa.

It is recognized that there are other forms of soil N that can also be analyzed (total Kjeldahl N, $NH_4$-N), and in this study these forms of N were also determined for comparison purposes in a more limited number of field tests. $NH_4$-N data was also

**TABLE 1**
**Average Postplanting (Within 7 Days Following Planting) Residual $NO_3$-N Determined on Air-Dried Samples Collected in the Upper 0.6 m of Soil in Lowest N Application Treatment Plots**

| | Soil $NO_3$-N in Upper 0.6 m Soil Profile (kg $NO_3$-N/ha) | | | | | | | |
|---|---|---|---|---|---|---|---|---|
| Year | Shafter REC | West Side REC | Merced County Site | Madera County Site | Fresno County Site | Tulare County Site | Kings County Site | Kern County Site |
| 1996 | 77 (6) | 166 (12) | 161 (14) | 171 (12) | 207 (23) | 141 (21) | 251 (23) | 150 (8) |
| 1997 | 62 (4) | 48 (3) | 157 (6) | 84 (10) | 53 (5) | 97 (14) | 121 (5) | 52 (7) |
| 1998 | 41 (4) | 61 (7) | 63 (2) | 115 (7) | 87 (10) | 67 (5) | 59 (3) | 66 (6) |
| 1999 | 51 (8) | 40 (5) | 122 (14) | 272 (19) | No site | 70 (6) | 61 (4) | 84 (11) |
| 2000 | 71 (11) | 40 (6) | 119 (9) | 161 (8) | 124 (7) | 78 (11) | 85 (9) | 71 (4) |

*Note*: Standard errors of the mean are shown in ( ) after the means.

variable across soils, test sites, and years, and $NH_4$-N levels were generally quite low relative to soil $NO_3$-N. Total N estimates by Kjeldahl measurements generally were in reasonable agreement (positive correlation [$r^2 = 0.71$] with relative soil $NO_3$-N levels at sites where specific comparisons were made, but were much more expensive and difficult to run).

## Long-Term Changes in Soil $NO_3$-N

Soil N as $NO_3$-N was converted into kg N/ha for each 0.3-m increment sampled in the soil profile during postplanting and postharvest sampling times. These data were combined with average soil bulk density measured at each field site, allowing calculation of net changes in soil test N as $NO_3$-N during the growing season (postplanting to postharvest). There are recognized limits in interpreting these data types, since values change over time with processes such as mineralization and denitrification. However, these changes in soil $NO_3$-N over time still represent a general index of soil changes in N status resulting from crop uptake and other processes/losses during the growing season.

Average changes in soil $NO_3$-N between the spring (planting time) and fall (postharvest) soil sample timings are given for some specific farm sites in 1997 as well as multisite averages for 4 of the 5 years of the study (Table 2). The 2000 soils data have not been completely analyzed and thus are omitted in this analy-

**TABLE 2**
**Average Changes in Soil $NO_3$-N Shown as the Postharvest (Fall) Sample Results Minus the Postplanting (Spring) Soil Test $NO_3$-N as a Function of N Application Treatments and Depths**

| | | Average Changes in Soil $NO_3$-N (Postharvest Minus Postplanting) (in kg N/ha as $NO_3$-N) | | | | | | | |
|---|---|---|---|---|---|---|---|---|---|
| | | 1997 Example Sites | | | | 7 or 8 Location Averages by Years | | | |
| Applied N Treatment (kg N/ha) | Depth in Soil Profile (m) | Shafter REC | Kern County | Tulare County | Kings County | 1996 (8 Sites) | 1997 (8 Sites) | 1998 (8 Sites) | 1999 (7 Sites) |
| 56 | 0–1.2 | –96 | –88 | –110 | –112 | –115 | –103 | –83 | –94 |
| | 1.2–2.4 | –8 | –2 | 4 | –3 | –6 | –14 | –18 | –30 |
| 112 | 0–1.2 | –69 | –66 | –75 | –115 | –86 | –88 | –92 | –81 |
| | 1.2–2.4 | 2 | –8 | –1 | –3 | 10 | –9 | 10 | –17 |
| 168 | 0–1.2 | –52 | –42 | –78 | –63 | –66 | –56 | –43 | –59 |
| | 1.2–2.4 | 0 | – 4 | 1 | 24 | 18 | 14 | 25 | 9 |
| 224 | 0–1.2 | –43 | –54 | –16 | –31 | –64 | –32 | –40 | –44 |
| | 1.2–2.4 | 8 | 6 | 26 | 31 | 29 | 25 | 40 | 39 |

*Note*: Data for specific 1997 study sites are as shown, while 1996 through 1999 averages were calculated across all study sites. Soil $NO_3$-N data is grouped as the soil surface to 1.2-m depth vs. the 1.2- to 2.4-m depth. Negative number (–) indicates net reduction in soil $NO_3$-N during the period from planting through postharvest.

sis. The negative numbers indicate a net “loss” or a reduction in soil $NO_3$-N content for all the upper 1.2-m depths in all N application treatments (56, 112, 168, and 224 kg N/ha treatments). This can be interpreted as mostly indicating net uptake of N from that zone of the soil (although other transformations can also account for some of the changes, and these were not measured). The positive numbers seen in the 1.2 to 2.4 m of the soil profile in the higher, applied N treatments potentially indicate there was more $NO_3$-N moved down into that deeper part of the soil profile during the course of the season. Again, other transformations can also account for part of the observed changes. If the cotton or subsequent crops cannot access this N source, it would be subject to leaching losses if moved further by water moving through the soil profile.

These data in general have indicated that most net depletion of soil $NO_3$-N (lower soil $NO_3$-N in the postharvest sampling than at postplanting) was seen in the upper 1.2 m of the soil profile. It could be argued that this depletion could result from leaching losses as well as denitrification, but the measured presence of significant root mass at depths down to 1.5 to 1.8 m at about one third of the sites over the 5 years indicated that plant uptake can be another reason for net depletion during the growing season even in the 1.2- to 2.4-m zone. Most other sites had root activity primarily in the upper 0.9 to 1.2 m of the profile. As levels of applied N increased at most sites, soil $NO_3$-N levels in the 1.2- to 2.4-m zone generally increased. More detailed analyses and discussion of dominant influences by site and year will be presented in future papers (in progress).

Irrigation water contributions to the N source available to the crop were monitored at all sites using monthly replicated water sampling and estimates of average water applications per irrigation. In general, most sites had relatively low irrigation water $NO_3$-N, as mountain snowmelt was a predominant irrigation water supply for many irrigation districts. Most of these sites had consistently less than 22 kg N/ha as $NO_3$-N per summer growing season that could be attributed to irrigation water sources; however, there were some sites with higher contributions from irrigation water N within each year. Two sites in 1996 had about 36 kg N/ha contributed by irrigation water $NO_3$-N, while one site received 28 kg N/ha, and another site 35 kg N/ha from irrigation water in 1997. Two sites in 1998 and three sites in 1999 had values ranging from 26 to 31 kg $NO_3$-N/ha attributable to the irrigation water. No research sites used in 2000 had irrigation water contributing more than 20 kg $NO_3$-N/ha during the summer irrigation season.

## Impact of Environmental Conditions/Year-on-Yields

Cotton yield response to N rates was affected by environmental conditions during the years when trials were conducted. Table 3 shows differences in heat unit and rainfall amounts and distribution for each study year. Lint yields in 1996 were moderate across all sites with a range of about 1100 lint/ha to 1700 kg lint/ha. In all but one of the field sites in 1996, there were no significant effects of N treatments on lint yields. (i.e., increasing N applications did not increase yields). Soil residual N in the upper 0.6 m as well as the lower profile were generally higher than in other years of the 5-year study.

In 1997, each location showing significant yield responses to increasing applied N had high lint yields (>1650 kg lint/ha), and planting-time soil $NO_3$-N levels in sites with lint yield responses were consistently low. In 1997, there were more locations with significant yield reductions at the lowest two N application rates (56 and 112 kg N/ha).

However, 1998 was a very difficult cotton production year, with poor weather during much of the season resulting in low yield potentials at most sites and in the state. Under reduced yield potential, less N was required for growth and yield, resulting in the expectation that responses to applied N would be fewer with moderate to high yields. In 1998, only two out of eight field sites showed significant yield responses to increases in applied N, and those yield responses were small.

Lint yields at most sites in 1999 and 2000 were moderate to high compared with 1998, resulting in a higher N demand for

**TABLE 3**
**2-Month Total Heat Units (Base 60°F) and Precipitation at the West Side Research and Extension Center Site for the Years 1996 Through 2000**

| | 2-Month Total Heat Units (Base 60° F) | | | | | | Total 2-Month Precipitation (cm) | | | | | |
|---|---|---|---|---|---|---|---|---|---|---|---|---|
| Year | Jan–Feb | Mar–April | May–June | July–Aug | Sept–Oct | Nov–Dec | Jan–Feb | Mar–April | May–June | July–Aug | Sept–Oct | Nov–Dec |
| 1996 | 13 | 140 | 680 | 1248 | 530 | 0 | 15.6 | 8.4 | 0.3 | 0 | 5.3 | 0 |
| 1997 | 4 | 178 | 777 | 1054 | 555 | 32 | 8.3 | 2.6 | 0.1 | 0 | 0.7 | 10.4 |
| 1998 | 1 | 97 | 364 | 1169 | 463 | 2 | 15.4 | 7.2 | 6.6 | 0 | 0.9 | 3.0 |
| 1999 | 1 | 79 | 550 | 958 | 666 | 12 | 5.2 | 4.0 | 0.1 | 0 | 0 | 0.16 |
| 2000 | 2 | 127 | 804 | 1001 | 480 | 0 | 8.9 | 5.2 | 0.3 | 0.4 | 4.6 | 0.9 |

*Note:* Heat units were calculated using the single triangle calculation approach using a base heat unit of 60°F and no upper threshold for heat-unit calculation. Temperature and precipitation measurements were made using equipment at an on-site weather station located approximately 0.2 km from the N experiment site.

growth and fruit production. In these final 2 years of the study, four out of seven sites (1999) and five out of eight sites (2000) showed significant yield responses to increasing applied N. However, only three of seven (1999) and two out of eight (2000) had significant yield responses to N applications in excess of 112 kg N/ha. The largest yield responses were from low-N plots at sites where spring residual soil $NO_3$-N was depleted (<60 kg N/ha in the upper 0.6 m of soil) due to repeated use of the same treatments over several consecutive years.

## Residual Soil $NO_3$-N Plus Applied N Relationship to Lint Yields

One of the primary goals of this study was to develop some basis for the use of soil residual $NO_3$-N levels as part of the decision process in estimating crop N application needs each year. The preplant or immediate postplanting soil samples from the upper 0.6 m of soil profile were selected as a minimal amount of soil sampling that would be easily collected and inexpensive enough to be accepted by growers and consultants. When all yield responses from all years and sites are regressed against residual $NO_3$-N in the upper 0.6 m of soil plus applied N (Fig. 1), it is evident that many factors in addition to N impact cotton lint yields across sites and years.

The large degree of scatter in the data is not reduced significantly by changing lint yield to relative lint yield (data not shown, in which treatment yields are expressed as a percent of yield in the lowest N treatment at each site). However, when the data were grouped according to ranges of soil $NO_3$-N levels in the upper 0.6 m of the soil profile within a week after planting, three levels were chosen to partition the data into sites differing in likelihood (probability) of crop responses to increasing levels of applied N. These levels chosen were: (1) less than 70 kg $NO_3$-N/ha in the upper 0.6 m of soil (Fig. 2); (2) between 70 and 125 kg $NO_3$-N/ha in the upper 0.6 m of soil (Fig. 3); and (3) over 125 kg $NO_3$-N/ha in the upper 0.6 m of soil (Fig. 4).

There are limits in interpreting only soil $NO_3^-$ data. It is recognized that there are other forms of N present in the soil, and that the relative mix of these forms changes in response to changing soil water status, temperature, and biological factors. However, differences in soil $NO_3$-N between sampling periods represent an index of changes in soil N status resulting from crop uptake and other processes that occur during the growing season. Although there are several forms of soil N, we chose to group the data using soil $NO_3^-$. Soil $NO_3^-$ is quickly and inexpensively measured as compared with other analyses, thus analyses of soil $NO_3^-$ could be readily available to growers from commercial soil-testing laboratories.

Some generalizations can be suggested with these groupings based on early-season soil $NO_3$-N that could be used to assess the likelihood of yield responses to applied N. When residual soil $NO_3$-N was less than 70 kg $NO_3$-N/ha in the upper 0.6 m of soil at planting, cotton yields increased significantly with increasing N applications in 13 of the 17 sites (Fig. 2) ($p < 0.05$). When planting time soil residual $NO_3$-N was between 70 and 125 kg $NO_3$-N/ha in the 0.6-m soil depth, yields were significantly affected ($p < 0.05$) by increasing N applications in 7 out of 11 sites (Fig. 3). Only 3 out of 11 sites showed significant ($p < 0.05$) yield increases to increasing applied N when residual soil $NO_3$-N exceeded 125 kg $NO_3$-N/ha in the upper 0.6 m of soil profile (Fig. 4). Growers and farm managers should understand that $NO_3$-N at planting time in the upper 0.6 m soil does not explain all available sources of N and will not provide as good an estimate of potentially available N as deeper soil sampling such as that done to a depth of 1.2 m. However, these ranges of planting-time $NO_3$-N could be used to improve grower confidence in reducing early fertilizer N applications by 25 to 50 kg N/ha when moderate (70 to 125 kg $NO_3$-N/ha) or higher (>125 kg $NO_3$-N/ha) soil N levels exist at planting time. Even if early-season N applications are reduced, growers will still have the option of using estimated fruit load and petiole $NO_3$-N data to determine if later supplemental N applications would be warranted.

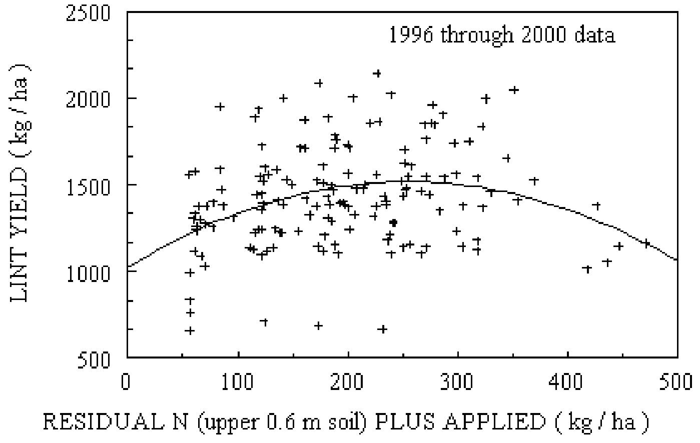

**FIGURE 1.** Lint yield (kg lint/ha) as a function of residual soil $NO_3$-N (top 0.6 m of soil profile) plus applied N (kg/ha), including all sites and years from 1996 to 2000. Curve fit is 2nd order polynomial, $r^2 = 0.328$.

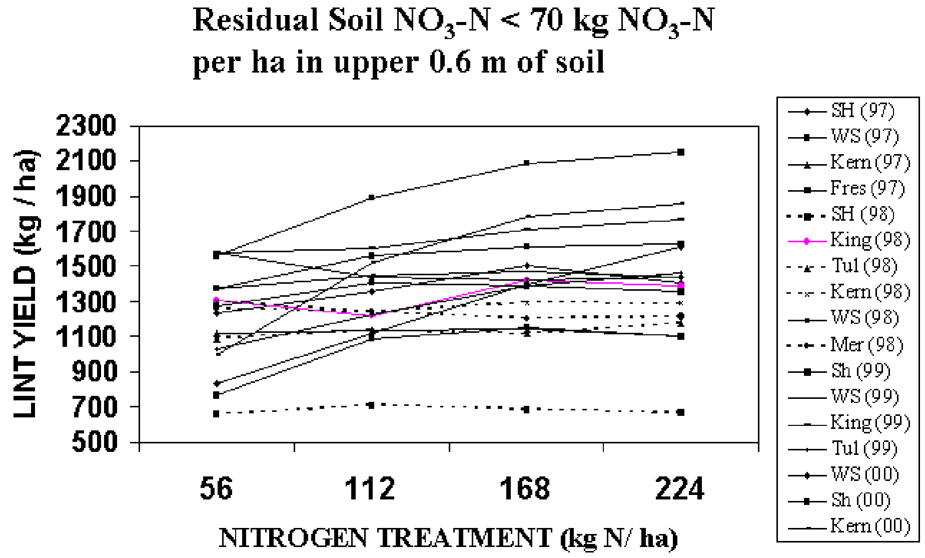

**FIGURE 2.** Lint yields as a function of N treatment for 17 sites where the lowest N treatment had residual soil $NO_3$-N less than 70 kg $NO_3$-N/ha in the upper 0.6 m of soil. Legend shows locations and years for sample sites that fit into this grouping. Sites for which there were significant differences ($p < 0.05$) in yields across N treatments are shown as solid lines, those with no significant differences between any means at different N levels are shown as dashed lines.

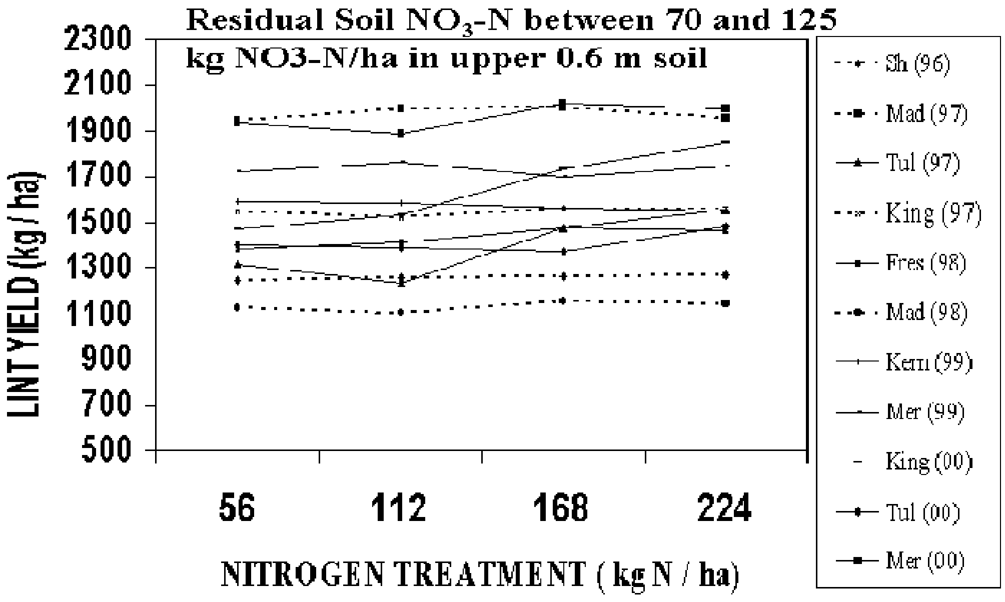

**FIGURE 3.** Lint yields as a function of N treatment for 11 sites where the lowest N treatment had a residual soil $NO_3$-N level between 70 and 125 kg $NO_3$-N/ha in the upper 0.6 m of soil. Legend shows locations and years for sample sites that fit into this grouping. Sites for which there were significant differences ($p < 0.05$) in yields across N treatments are shown as solid lines, those with no significant differences between any means at different N levels are shown as dashed lines.

## CONCLUSIONS

Growers trying to maximize yields and financial returns during difficult economic times are reluctant to reduce relatively inexpensive fertilizer applications and risk yield losses due to N deficiencies. In attempting to reduce applied N to better use residual N reserves in the soil, growers will need to use information concerning cropping history, measurements of soil N, and possibly in-season measurements of crop N status such as petiole $NO_3^-$ analysis. From a management standpoint, it makes sense

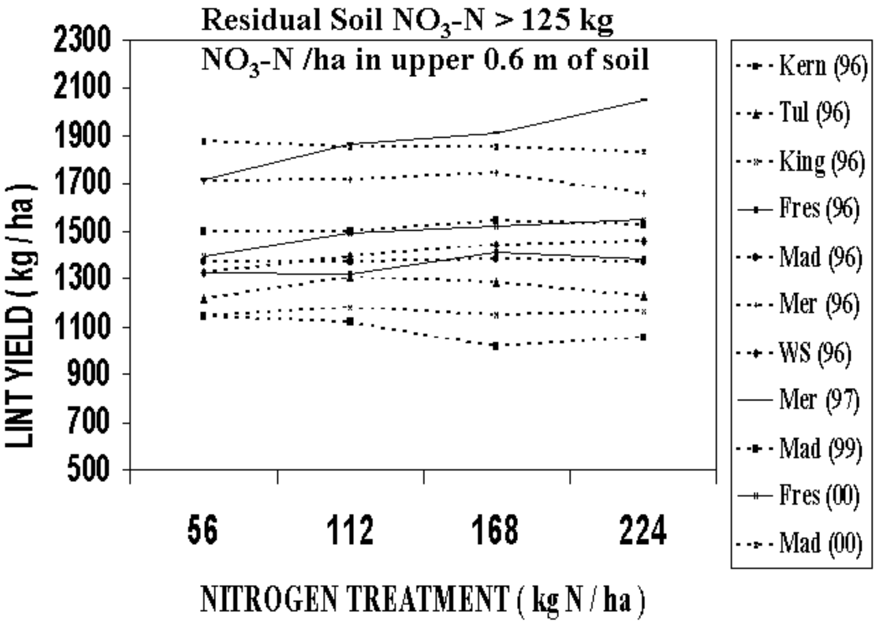

**FIGURE 4.** Lint yields as a function of N treatment for 11 sites where the lowest N treatment had a residual soil $NO_3$-N level greater than 125 kg $NO_3$-N/ha in the upper 0.6 m of soil. Legend shows locations and years for sample sites that fit into this grouping. Sites for which there were significant differences ($p < 0.05$) in yields across N treatments are shown as solid lines, those with no significant differences between any means at different N levels are shown as dashed lines.

for farm managers to measure soil N status, adjust N application rates, and monitor in-season plant N status. Economically, however, the common perception is that cost of management time and analytical services may not represent a saving compared with at-planting application of an additional increment of inorganic N fertilizers.

Plant N uptake data collected at selected sites in 3 years of this experiment (data not shown) essentially confirms earlier work in California and Israel, which indicated the need for 115 to 135 kg N per 1000 kg of cotton lint produced[7,8]. These results are important considering the lack of yield response noted in many of the reported study sites across a wide range of applied N. The results of the current study do not indicate that only 56 or 112 kg N/ha are needed to produce moderate to high cotton yields (1100 to 1800 kg lint/ha or more), but rather indicate that soil residual N (from various forms) can serve as a major additional source of N in meeting crop N requirements. A planting-time soil $NO_3$-N could be used as a decision aid in assessing likely cotton lint yield responses to supplemental N, providing more security in making decisions of whether or not to reduce early-season fertilizer N applications.

## ACKNOWLEDGMENTS

The assistance and generosity of growers/cooperators in these studies is gratefully acknowledged. Financial support for this project has been provided by the California State Support Committee in combination with core funds from Cotton Incorporated. Funding for some related projects on plant N nutrition that has helped fund parts of this project has come from the California Department of Food and Agriculture, Fertilizer Research and Education program, the University of California Cooperative Extension, and California Crop Improvement Association.

| | | |
|---|---|---|
| Received: | July | 23, 2001 |
| Revised: | October | 12, 2001 |
| Accepted: | October | 29, 2001 |
| Published: | November | 20, 2001 |
